# A co-twin-control study of altered sensory processing in autism

**DOI:** 10.1177/1362361321991255

**Published:** 2021-03-01

**Authors:** Janina Neufeld, Mark J Taylor, Karl Lundin Remnélius, Johan Isaksson, Paul Lichtenstein, Sven Bölte

**Affiliations:** 1 Karolinska Institutet, Sweden; 2 Region Stockholm, Sweden; 3 Uppsala University, Sweden; 4 Curtin University, Australia

**Keywords:** autism spectrum disorders, behavioral genetics, environmental factors, sensory processing, sensory profile, twin design

## Abstract

Autism spectrum disorder is associated with sensory processing alterations, such as sensory hyper- and hypo-responsiveness. Twin studies are scarce in this field, but they are necessary in order to disentangle the genetic and environmental contributions to this association. Furthermore, it is unclear how different neurodevelopmental/psychiatric conditions contribute to altering sensory processing. We investigated the association between autistic traits/autism spectrum disorder diagnosis and sensory processing alterations in twins (*N* = 269), using the adult/adolescent sensory profile, which differentiates four sub-domains: Low Registration, Sensation Seeking, Sensory Sensitivity, and Sensation Avoiding. While the associations between autistic traits and *Low Registration* and *Sensation Avoiding* persisted within monozygotic (genetically identical) twins, *Sensory Sensitivity* was only associated with autistic traits within dizygotic twins. In multivariate analyses with different neurodevelopmental/psychiatric diagnoses as predictor variables, autism spectrum disorder and attention deficit hyperactivity disorder were the strongest predictors for two adult/adolescent sensory profile sub-domains each. The results suggest that the association between autistic traits and *Sensory Sensitivity* is influenced by genetics while non-shared environmental factors influence the associations between autistic traits and *Low Registration* and *Sensation Avoiding*. They further indicate that altered sensory processing is not specific to autism spectrum disorder, while autism spectrum disorder is a strong predictor of certain sensory processing alterations, even when controlling for other (comorbid) neurodevelopmental/psychiatric conditions.

**Lay abstract:**

Individuals diagnosed with autism often describe that they process sensory information differently from others, and many experience sensory issues as problematic. For instance, an increased sensitivity to smells or sounds can make participating in social settings challenging. While sensory issues are now part of the diagnostic criteria for autism, they also co-occur with other psychiatric diagnoses such as attention deficit hyperactivity disorder and anxiety disorders. It is unclear to what extent the relationship between autism and alterations in sensory processing are due to genetics or environment. In addition, more research is needed on how autism, as compared to other diagnoses, is associated with sensory issues. Using a twin study, we found that genetic factors influenced self-reported reactivity to sensory stimuli in autism while environmental factors influenced other sensory issues (e.g. difficulties in detecting or differentiating sensory input). Hence, sensory hyper-reactivity might be an early onset core feature of autism, while other domains of alterations in sensory processing might develop later, influenced by the environment. Moreover, autism was more strongly associated with sensory issues related to increased sensitivity/reactivity as compared to other psychiatric diagnoses. However, attention deficit hyperactivity disorder was more strongly related to deficits in detecting/differentiating sensory stimuli and with an increased drive to seek sensory input. Our results indicate that sensory issues are not specific to autism, but that some aspects of altered sensory processing are more relevant for autism than for other diagnoses.

## Introduction

Atypical sensory processing, such as hyper- or hypo-responsiveness to sensory stimuli or atypical sensory interests, has been reported to occur in 69%–95% of individuals diagnosed with autism spectrum disorder (ASD; Hazen et al., 2014; Posar & Visconti, 2018; Robertson & Baron-Cohen, 2017). Today, they are conceptualized as a defining criterion of ASD, forming part of the criteria in the repetitive behavior/restricted interests domain (*Diagnostic and Statistical Manual of Mental Disorders* 5th ed.; DSM-5; American Psychiatric Association, 2013). Sensory processing alterations can be defined as unusual perception of or reaction to sensory stimuli, including both hyper- and hypo-responsiveness to sensory input (Posar & Visconti, 2018). Within the DSM-5, sensory hyper-responsiveness is described as “aversive response” to sensory inputs that are commonly tolerated, while hypo-responsiveness is described as “apparent indifference” to stimuli commonly inducing a reaction (American Psychiatric Association, 2013).

Atypical sensory processing has been suggested to precede and influence the atypical social cognition characterizing ASD (Robertson & Baron-Cohen, 2017) and can interfere with daily living requirements (Dellapiazza, 2018). Importantly, sensory processing challenges are experienced by many individuals on the autism spectrum as the true core of their condition (Chamak et al., 2008). Therefore, understanding the etiology of sensory processing alterations is crucial for deciphering ASD and the challenges in daily living associated with it. Since ASD is likely to form the extreme end of a continuum of autistic traits approximating a normal distribution across the general population (Colvert et al., 2015), investigating the relationship between sensory processing alterations and quantitative autistic traits seems more informative than solely using a categorical clinical view, comparing people with and without ASD diagnosis. Positive correlations between autistic traits and self- or parent-rated sensory processing alterations have been found in typically developed (TD) adults and children, and adults diagnosed with ASD, suggesting a linear relationship (Hilton et al., 2007; Horder et al., 2014; Robertson & Simmons, 2013; Tavassoli et al., 2014).

The etiology of sensory processing alterations in ASD remains poorly understood. Family studies indicate that sensory processing alterations in individuals with ASD are at least partly shared by their family members (De la Marche et al., 2012; Donaldson et al., 2017; Glod et al., 2017; Hilton et al., 2016; Uljarević et al., 2014). In order to disentangle genetic from environmental influences, twin studies are needed. Since monozygotic (MZ) twins share almost all genes while dizygotic (DZ) twins share on average half of their segregating genes, inferences on genetic and environmental influences can be made by comparing MZ and DZ twins. Recently, a population-based twin study suggested that sensory hyper-reactivity is largely heritable and that most of its association with ASD can be explained by common genetics (Taylor et al., 2018). The study was limited, however, by the use of a brief, five-item assessment of sensory reactivity, not allowing for the investigation of sub-domains of sensory processing alterations.

Sensory processing alterations are not specific to ASD (Posar & Visconti, 2018), but co-occur also within other neurodevelopmental and psychiatric conditions, including attention deficit hyperactivity disorder (ADHD) and anxiety disorders (Ghanizadeh, 2011; Lane et al., 2012; Liss et al., 2005). Since about 70% of the individuals diagnosed with ASD show one or more neuropsychiatric comorbidities (Rosen et al., 2018; Simonoff et al., 2008), co-existing conditions (which were often not assessed or reported in studies) might have confounded the association between ASD and altered sensory processing in previous research.

Here, we investigated the genetic and environmental impact on the relationships between sensory processing alterations and both continuous autistic traits and categorical ASD diagnosis, utilizing a co-twin-control design in a sample of carefully characterized MZ and DZ twins, enriched for ASD, ADHD, and other neurodevelopmental and psychiatric conditions. Sensory processing alterations were assessed with the adult/adolescent sensory profile (AASP), distinguishing four sub-domains of sensory hyper- and hypo-responsiveness. Finally, we explored the specificity of the co-occurrence of sensory processing alterations with ASD by modeling also other neurodevelopmental and psychiatric conditions within the same model.

## Methods

### Participants

The twins were assessed within the ongoing “Roots of Autism and ADHD Twin Study in Sweden” (RATSS; Bölte et al., 2014), a project closely associated with the population-based “Child and Adolescent Twin Study in Sweden” (CATSS; Anckarsäter et al., 2011). In CATSS, twins are screened with the “Autism-Tics, ADHD and other Comorbidities inventory” (A-TAC; Hansson et al., 2005) and primarily twin pairs (and triplets) exhibiting discordancy or concordancy for ASD or ADHD-like traits on the A-TAC are recruited for in-depth assessment in RATSS. This approach increases the sensitivity for assessing within-pair associations with ASD or ADHD. In addition, twins with low trait levels are recruited in order to cover the entire spectrum of autistic traits. Zygosity was determined on a panel of 48 single nucleotide polymorphisms (Hannelius et al., 2007; ~85% of pairs) or a four-item zygosity questionnaire. Four pairs where the zygosity was not determined at the time of analysis were excluded. We further excluded nine opposite sex twin pairs, one different sex individual from a triplet, one pair from a family with two twin pairs, and 33 twin pairs with missing data (i.e. more than two items per sub-domain missing on the AASP or missing autistic traits data—in at least one twin). The included sample consisted of 133 twin pairs and one triplet. Of these 269 individuals, 60 were diagnosed with ASD, further 84 had one or more other diagnoses (ADHD, tic disorders, or other neurodevelopmental disorders (NDDs), anxiety disorders, or depression) and the remaining 125 individuals did not fulfill such diagnostic criteria (see Table 1 for a summary of sample characteristics). Thirty-four twin pairs were discordant (MZ/DZ = 16/18) and 13 twin pairs were concordant (MZ/DZ = 9/4) for ASD diagnosis.

**Table 1.** Sample characteristics.

Measure	Whole sample	MZ	DZ	MZ vs DZ test statistic/*p*
Total (*N*)	269	166	103	
ASD (*N*)	60	34	26	8142 / 0.36
ADHD (*N*)	**68**	**33**	**35**	**7344 / 0.01**
Other NDDs (*N*)	44	25	19	8260 / 0.47
Affective disorders (*N*)	51	27	24	7948 / 0.15
Sex (m/f)	149 / 120	90/76	59/44	8811 / 0.62
Age range (years)	10–31	10–29	10–31	–
Mean age (SD)	17.7 (5.5)	18.1 (5.5)	17.0 (5.3)	1.73 / 0.09
Mean SRS-2 (SD)	**40.2 (32.6)**	**35.4 (31.5)**	**48.0 (32.9)**	**−3.10 / 2.2e** ^**−3**^
Mean Δ SRS-2 (SD)	**22.7 (25.4)**	**17.5 (21.8)**	**31.8 (28.5)**	**−3.04 / 3.2e**−3****
Mean IQ (SD)	97.8 (15.9)	98.5 (16.8)	96.6 (14.3)	0.96 / 0.33
Mean Δ IQ (SD)	**10.6 (9.6)**	**8.8 (9.0)**	**13.7 (9.8)**	**−2.90 / 4.6e^**−3**^**

MZ: monozygotic; DZ: dizygotic; ASD: autism spectrum disorder; ADHD: attention deficit hyperactivity disorder; NDD: neurodevelopmental disorder; SRS-2: Social Responsiveness Scale–Second Edition ; SD: standard deviation; IQ: intelligence quotient.

Other NDDs: neurodevelopmental disorders other than ASD or ADHD; affective disorders: diagnosed with depression or anxiety disorder; Δ: difference between twins of the same pair.

Zygosity groups were compared using the Wilcoxon sum rank tests (binary measures) or *t*-tests (continuous measures). Significant zygosity group differences (uncorrected) are printed in bold black.

#### Socioeconomic background

Participants distributed as follows among five categories of economic background: households where the total income per month in Swedish crowns (10 Swedish crowns = approximately US$1.1) before tax was below 20.000 (7.5%), 20.000–40.000 (21.5%), 40.000–60.000 (34%), 60.000–80.000 (14.5%), or above 80.000 (10%). For the remaining 12.5% of participants, the parents did not report their income. Furthermore, 44.6% of the participants’ parents reported to have completed senior high school and an additional 32.2% further studied at a university for at least 3 years, while 8.6% of parents reported that their highest education was the completion of primary school, 6.1% reported not to have completed primary school, and 8.6% did not respond to this question. Ethnicity was assessed after the initial data collection in 57% of the participants’ parents. Of these, 95% reported to have a European ethnic background.

### Ethical considerations

Informed consent was obtained from all participants or their caregivers and ethical approval for the study was given by the Regional Ethical Review Board in Stockholm.

### Autistic traits, ASD, and other diagnoses

Autistic traits were assessed using the Swedish versions of the parent-reported Social Responsiveness Scale–Second Edition (SRS-2) for children or adults, and total raw scores were used as recommended for research settings (Constantino et al., 2003; Constantino & Gruber, 2005, 2019). The SRS-2 consists of 65 items rated on a Likert-type scale, focusing on the individual’s behavior during the past six months, with higher scores indicating higher autistic traits (maximum score = 195) and individuals with ASD typically scoring between 60 and 165 (Constantino et al., 2003). The SRS has demonstrated good to excellent psychometric properties for test–retest reliability (0.80–0.97), inter-rater reliability (0.75–0.95), and satisfactory convergent validity (0.35–0.58) with the “Autism Diagnostic Observation Schedule” (ADOS) and the “Autism Diagnostic Interview–Revised” (ADI-R; Bölte et al., 2008; Constantino et al., 2003). The twins were assessed by a team of experienced clinicians, and a consensus ASD diagnosis was supported by medical history in addition to a set of gold standard diagnostic tools that included the ADI-R (Rutter et al., 2003) and the ADOS, second version (Lord et al., 2012).

Other NDDs such as ADHD, tic disorders, specific learning disorders and intellectual disability, and psychiatric disorders such as depression and anxiety were determined based on a multitude of information sources. These included results from the “Kiddie Schedule for Affective Disorders and Schizophrenia” (K-SADS; Kaufman et al., 1997), the “Diagnostic Interview for ADHD in adults” (Kooij & Francken, 2010) and the “Structured Clinical Interview for DSM-IV” (SCID, axis I). General cognitive abilities were assessed using the Wechsler Intelligence Scales for Children or Adults–Fourth Edition (WISC-IV/WAIS-IV; Wechsler, 2003, 2008).

### Sensory processing alterations

Alterations in sensory processing were estimated using the “Adult/Adolescent Sensory Profile” (AASP), a self-report measure assessing sensory processing alterations across several sensory modalities and differentiating four sub-domains (*Low Registration, Sensory Sensitivity, Sensation Seeking*, and *Sensation Avoiding*; Brown & Dunn, 2002). The Swedish version was used, which is adapted to Swedish conditions both linguistically and culturally and validated in 500 individuals between 11 and 65 years (Brown & Dunn, 2014). The *Low Registration* sub-domain contains questions assessing how commonly individuals do not notice sensory stimuli or are unable to detect them such as “I don’t smell things that other people say they smell.” The *Sensory Sensitivity* sub-domain contains questions concerning aversive effects of sensory stimuli, such as “I am distracted if there is a lot of noises around.” The *Sensation Seeking* sub-domain assesses actions that people undertake in order to enhance sensory input, for example, “I like to go to places that have bright lights and that are colorful.” In contrast, the *Sensation Avoiding* sub-domain assesses actions that are undertaken in order to limit sensory input, for example, “I stay away from noisy settings.” Higher scores indicate more sensory symptoms and individuals diagnosed with ASD typically score higher than TD individuals in the domains *Low Registration, Sensory Sensitivity*, and *Sensation Avoiding*, but often lower in the *Sensation Seeking* domain of the AASP (Chien et al., 2017; Crane et al., 2009).

### Statistical analyses

We investigated the associations between the four AASP sub-domains and both continuous autistic traits and categorical ASD diagnosis using linear regressions within the Generalized Estimating Equations (GEE) framework with doubly robust standard errors (drgee package) (Zetterqvist & Sjölander, 2015). This approach does not assume normally distributed variables and has been employed in previous studies of our lab, for instance, to investigate the within-twin pair associations between autistic traits and cognitive test performance or brain connectivity (Neufeld et al., 2018, 2020).

First, linear regressions were conducted across individuals, treating twins as individuals, but adjusting the standard errors for twin clustering, with either autistic traits or ASD diagnosis as main predictor, both unadjusted and adjusted for sex and age. This first model is more similar to a regression in a non-twin cohort and hence more comparable to regression analyses reported in the literature; however, the outcomes need to be interpreted with caution since they might be influenced by our sampling method (primarily selecting twin pairs where at least one twin had elevated autistic traits).

The main analysis was the next step, where we performed *conditional* linear regressions within-twin pairs with autistic traits as main predictor, modeling separate estimates for MZ and DZ sub-cohorts. This approach has been recommended in order to assess within-pair associations (Neuhaus & McCulloch, 2006) and been utilized previously, both by us (e.g. Cauvet et al., 2020; Neufeld et al., 2018, 2020) and others (see, for example, D’Onofrio et al., 2020). This second model investigates the same associations, while implicitly adjusting for all shared familial factors, including different degrees of genetics (50% in DZ and 100% in MZ twins). Age and sex, which are explicitly modeled in the first model, are implicitly accounted for in the within-pair model, since twins of each pair were investigated at the same time and we only included same sex pairs in this study. Since non-shared environmental factors are, except for post-twinning mutations, the only type of factors making MZ twins dissimilar from each other, associations that are observed within MZ twins are immanently determined by non-shared environment. Within DZ twins, associations can be driven by both non-shared environment and genetics. Therefore, comparing the estimates from MZ and DZ within-pair analysis allows drawing conclusion regarding the presence of genetic effects on an association, although no quantitative estimation of the latter. For this, zygosity group–specific estimates were then compared using a Wald-type *χ*^2^ test statistic. Finally, we re-ran the adjusted models across individuals while including further binary predictors aside from ASD. These were ADHD, NDDs other than ASD or ADHD (e.g. communication disorders, specific learning disorders, or motor disorders), and affective disorders (depression and anxiety). These binary predictors, indicating presence or absence of a clinical diagnosis, were not exclusive, as owing to their co-occurrence (comorbidity).

Since each of the AASP sub-domains served as outcome in a separate model, the Bonferroni corrected alpha-level (at *α* = 0.05) was set to *p* = 0.0125 (0.05/4) in order to account for multiple comparisons (four associations tested). Because the different steps (adjusted and unadjusted, across the cohort and within-pairs, using autistic traits or ASD diagnosis as measure for ASD) had the purpose to further explore and verify the same associations between ASD and the four sensory processing sub-domains, we did not additionally correct for the amount of follow-up models.

## Results

### Association between sensory processing alterations and ASD—across individuals and within-pairs

**Figure 1.**
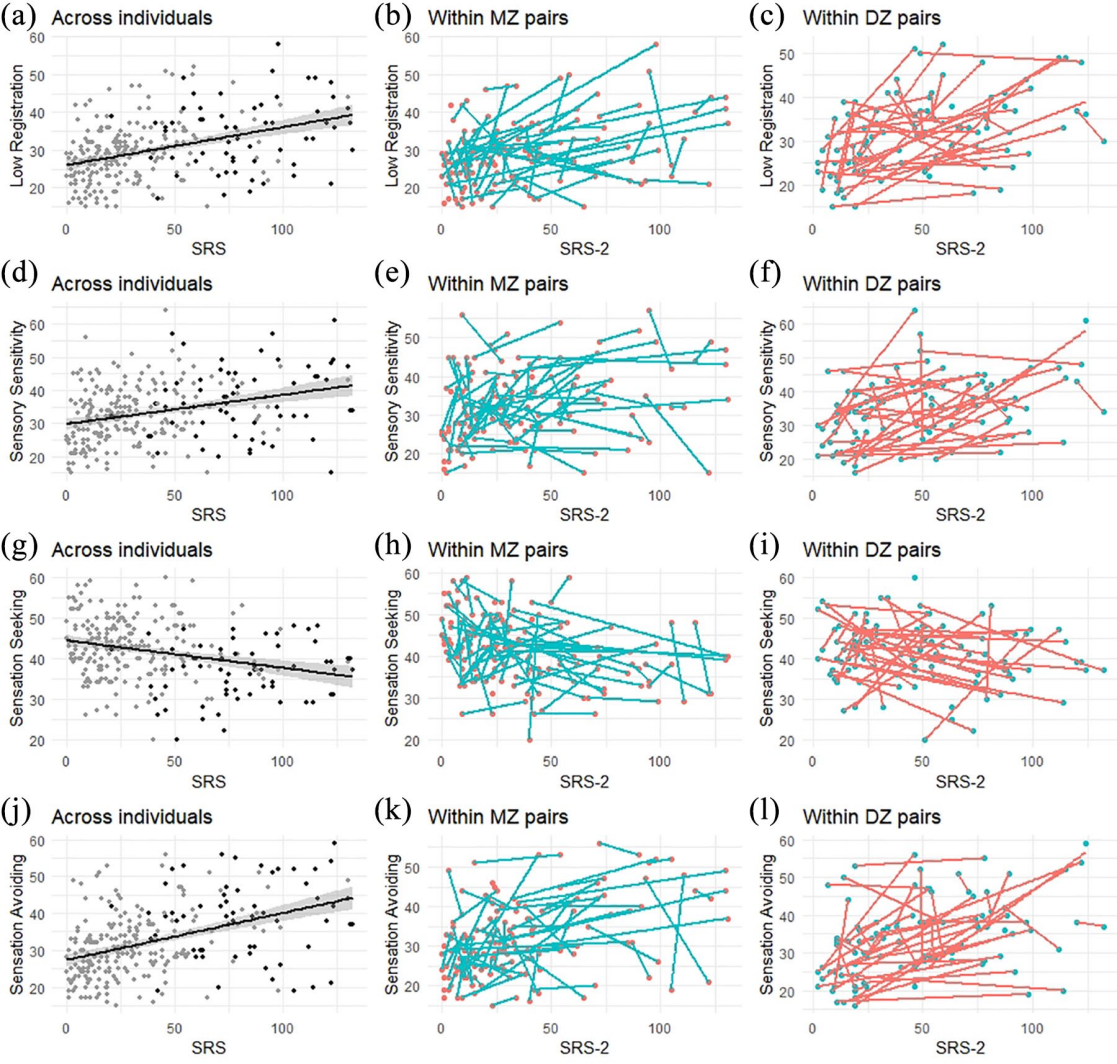
Visualization of the unadjusted regression results. As an approximation to visualize the regression results, the four AASP quadrants (one per row) are plotted as a function of autistic traits (SRS-2). The left most column represents the unadjusted models across individuals (a, d, g, and j), where black dots represent the individuals diagnosed with ASD and gray dots those without ASD. The middle column represents the within MZ associations where twins of a pair are connected with blue lines (b, e, h, and k). The right most column represents the within DZ associations where twins of each pair are connected with a red line (c, f, i, and l).

The results are summarized below and shown in detail in Tables 2 to 4. Figure 1(a) to (l) visualizes the outcomes of the unadjusted across cohort associations and within MZ and DZ associations between autistic traits and the four sensory processing sub-domains ((a) to (c) = Low Registration, (d) to (f) = Sensory Sensitivity, (g) to (i) = Sensation Seeking, and (j) to (l) = Sensation Avoiding).

**Table 2.** Results from linear regressions across individuals and within-twin pairs with autistic traits as main predictor of sensory processing alterations.

Model	Effect of autistic traits			MZ vs DZ
	*b* (95% CI)	SE	*Z*/*p*	*χ*^2^/*p*
Low Registration—				
Across unadjusted	**0.10 (0.07, 0.13)**	**0.02**	**6.11 / 9.9e^−10^**	0.04 / 0.85
Across adjusted	**0.09 (0.06, 0.13)**	**0.02**	**5.55 / 2.9e^−8^**	
Within-pairs (MZ)	**0.12 (0.07, 0.17)**	**0.03**	**4.42 / 2.5e^−6^**	
Within-pairs (DZ)	**0.13 (0.06, 0.20)**	**0.04**	**3.65 / 2.8e^−4^**	
Sensory Sensitivity—				
Across unadjusted	**0.09 (0.05, 0.13)**	**0**.**02**	**4.59 / 4.4e^^−6^^**	**4.23 / 0.04**
Across adjusted	**0.10 (0.06, 0.13)**	**0.02**	**4.84 / 1.3e^−6^**	
Within-pairs (MZ)	0.05 (−0.01, 0.10)	0.03	1.68 / 0.09	
Within-pairs (DZ)	**0.15 (0.07, 0.22)**	**0.04**	**3.74 / 2.0e^−4^**	
Sensation Seeking—				
Across unadjusted	**−0.07 (−0.09, −0.04)**	**0.01**	**−5.41 / 6.4e^−8^**	0.10 / 0.75
Across adjusted	**−0.06 (−0.09, −0.03)**	**0.01**	**−4.35 / 1.4e^−5^**	
Within-pairs (MZ)	−0.06 (−0.11, 0.0)	0.03	−2.03 / 0.04	
Within-pairs (DZ)	−0.04 (−0.10, 0.02)	0.03	−1.35 / 0.18	
Sensation Avoiding—				
Across unadjusted	**0.13 (0.09, 0.16)**	**0.02**	**7.02 / 2.2e^−12^**	2.51 / 0.11
Across adjusted	**0.14 (0.10, 0.18)**	**0.02**	**7.55 / 4.5e^**−14**^**	
Within-pairs (MZ)	**0.08 (0.03, 0.12)**	**0.02**	**3.16 / 1.6e^−3^**	
Within-pairs (DZ)	**0.15 (0.07, 0.23)**	**0.04**	**3.75 / 2.0e^−4^**	

MZ: monozygotic, *N* = 166; DZ: dizygotic, *N* = 103; *b*: regression coefficient; CI: confidence interval; SE: standard error; *Z: Z* statistics; across: across individuals (treating twins as individuals adjusting for twin clustering), *N* = 269; adjusted: adjusted for sex and age across individuals.

The covariate outcomes are presented in Supplementary Table 2.

Significant outcomes are printed in bold black.

**Table 3.** Results from linear regressions across individuals with ASD diagnosis as main predictor of sensory processing alterations.

Model	*b* (95% CI)	SE	*Z*/*p*
Low Registration			
Unadjusted	**5.32 (2.80, 7.85)**	**1.29**	**4.13 / 3.6e−5**
Adjusted	**4.86 (2.39, 7.33)**	**1.26**	**3.85 / 1.2e−4**
Sensory Sensitivity			
Unadjusted	**4.28 (1.05, 7.51)**	**1.65**	**2.60 / 0.01**
Adjusted	**4.33 (1.23, 7.42)**	**1.58**	**2.74 / 0.01**
Sensation Seeking			
Unadjusted	**−6.56 (−8.53, −4.59)**	**1.00**	**−6.54 / 6.2e−11**
Adjusted	**−6.18 (−8.19, −4.16)**	**1.03**	**−6.01 / 1.9e−9**
Sensation Avoiding			
Unadjusted	**8.05 (4.91, 11.18)**	**1.60**	**5.03 / 4.8e−7**
Adjusted	**8.17 (5.16, 11.18)**	**1.54**	**5.32 / 1.0e−7**

ASD: autism spectrum disorder; *b*: regression coefficient; CI: confidence interval; SE: standard error; *Z: Z* statistics.

Results across individuals (treating twins as individuals), *N* = 269; adjusted: adjusted for sex and age.

The covariate outcomes are presented in Supplementary Table 2.

Significant outcomes are printed in bold black.

**Table 4.** Results from linear regressions across individuals with different diagnosis categories.

Model	ASD *b* (95% CI)/SE/*p*	ADHD *b* (95% CI)/SE/*p*	Other NDDs *b* (95% CI)/SE/*p*	Affective *b* (95% CI)/SE/*p*
(a) All diagnoses as predictors of sensory processing in the same model				
Low Registration	**3.39 (0.96, 5.82) / 1.24 / 0.01**	**4.89 (2.00, 7.78) / 1.47 / 9.2e−4**	0.76 (−1.69, 3.21) / 1.25 / 0.55	2.11 (−0.52, 4.73) / 1.34 / 0.12
Sensory Sensitivity	2.09 (−1.06, 5.25) / 1.61 / 0.19	**5.98 (3.32, 8.64) / 1.36 / 1.1e−5**	1.70 (−1.04, 4.43) / 1.40 / 0.23	**5.68 (2.73, 8.62) / 1.50 / 1.6e−4**
Sensation Seeking	**−6.69 (−8.72, −4.67) / 1.03 / 4.5e−11**	1.78 (−0.64, 4.20) / 1.23 / 0.15	0.72 (−1.91, 3.35) / 1.34 / 0.59	−0.47 (−2.58, 1.64) / 1.08 / 0.66
Sensation Avoiding	**6.25 (2.99, 9.52) / 1.66 / 1.7e−4**	**3.65 (1.15, 6.15) / 1.28 / 4.3e−3**	3.16 (0.10, 6.21) / 1.56 / 0.04	**4.82 (2.16, 7.48) / 1.36 / 3.8e−4**
(b) Each diagnosis as predictors of sensory processing in a separate model				
Low Registration	**4.86 (2.39, 7.33) / 1.26 / 1.2e−4**	**6.15 (3.39, 8.92) / 1.41 / 1.3e−5**	2.87 (0.38, 5.35) / 1.27 / 0.02	3.39 (0.62, 6.16) / 1.41 / 0.02
Sensory Sensitivity	**4.33 (1.23, 7.42) / 1.58 / 0.01**	**7.68 (4.87, 10.49) / 1.43 / 8.1e−8**	**3.85 (0.98, 7.18) / 1.57 / 0.01**	**7.05 (3.93, 10.17) / 1.59 / 9.5e−6**
Sensation Seeking	**−6.18 (−8.19, −4.16) / 1.03 / 1.9e−9**	0.28 (−2.02, 2.59) / 1.18 / 0.81	−0.61 (−3.33, 1.39) / 1.25 / 0.63	−0.84 (−3.02, 1.34) / 1.11 / 0.45
Sensation Avoiding	**8.17 (5.16, 11.18) / 1.54 / 1.0e−7**	**6.46 (3.72, 9.20) / 1.40 / 3.8e−6**	**5.81 (2.54, 9.08) / 1.67 / 5.0e−4**	**6.27 (3.48, 9.05) / 1.42 / 1.0e−5**

ASD: autism spectrum disorder; CI: confidence interval; SE: standard error; ADHD: attention deficit hyperactivity disorder.

Different diagnostic categories (ASD, ADHD, other NDDs, and affective disorders) as predictors of sensory processing alterations (a) within a joint model or (b) within separate models. Other NDDs: neurodevelopmental disorders other than ASD or ADHD; affective disorders: depression or anxiety disorders. All models were adjusted for sex and age.

Significant outcomes are printed in bold black.

#### Low Registration

Both higher autistic traits and ASD diagnosis predicted increased *Low Registration* scores across individuals in both unadjusted and adjusted models. Autistic traits predicted also increased *Low Registration* similarly within both MZ and DZ twin pairs.

#### Sensory Sensitivity

Across individuals, both higher autistic traits and ASD diagnosis predicted increased *Sensory Sensitivity*, similarly in unadjusted and adjusted models. The association with autistic traits remained within DZ but not within MZ twin pairs, and this zygosity group difference was significant.

#### Sensation Seeking

In contrast to all other AASP sub-domains, higher autistic traits and ASD diagnosis predicted reduced *Sensation Seeking* across individuals in unadjusted and adjusted models. The relationship between autistic traits and *Sensation Seeking* was not significant within MZ or DZ twin pairs.

#### Sensation Avoiding

Both higher autistic traits and ASD diagnosis predicted more *Sensation Avoiding* across individuals in unadjusted and adjusted models, as did autistic traits similarly within both MZ and DZ twin pairs.

### Sex and age effects

Across individuals, where sex and age were modeled explicitly, female sex predicted higher *Low Registration (model with autistic traits: b (95% CI)* *=* *2.68 (0.61, 4.75), standard error (SE)* *=* *1.06, p* *=* *0.01; model with ASD diagnosis: b (95% CI)* *= 2.71 (0.59, 4.83) SE = 1.08, p = 0.01)* and *Sensory Sensitivity* scores *(model with autistic traits: b (95% CI) = 4.42 (2.08, 6.76), SE = 1.20, p = 2.2e^−4^; model with ASD diagnosis: b (95% CI) = 4.44 (2.01, 6.88), SE = 1.24, p = 3.5e^−4^)*. Age was negatively associated with *Low Registration* in the model with ASD diagnosis *(b (95% CI) = −0.33 (−0.52, −0.15), SE = 0.09, p = 3.3e^−4^)*. Complete results for the covariates (both for the models with autistic traits and ASD diagnosis) are shown in Supplementary Table 2.

### Sensory processing alterations in association with ASD adjusted for other diagnoses

When ADHD, other NDDs, and affective disorders were included in addition to ASD diagnosis, sex and age as predictors of sensory processing alterations across individuals, the associations between ASD diagnosis and *Low Registration* and *Sensory Sensitivity*, respectively, were lost (see (a) in Table 4). In contrast, ADHD was associated with both these AASP sub-domains while affective problems predicted only increased *Sensory Sensitivity.* However, ASD was the only diagnosis that was (negatively) associated with *Sensation Seeking* symptoms and the strongest predictor of increased *Sensation Avoiding*, followed by affective disorders and ADHD. Modeling each diagnosis separately, other NDD diagnosis also predicted increased *Sensory Sensitivity* and several associations were stronger (larger estimates and smaller *p*-values) than in the model including the four diagnostic categories (see (b) in Table 4).

## Discussion

In this co-twin-control study, we investigated the relationships between parent-reported continuous autistic traits, categorical clinical ASD diagnosis, and self-rated alterations in sensory processing. Both continuous and categorical ASD similarly predicted sensory processing alterations across individuals. Associations between autistic traits and both *Low Registration* and *Sensation Avoiding* were also found within MZ twins, suggesting non-shared environmental influences. In contrast, the association between autistic traits and *Sensory Sensitivity* was stronger and only significant within DZ twins, indicating a genetic effect for this sub-domain. In addition, we assessed whether altered sensory processing was specifically associated with ASD as compared to other NDDs or psychiatric disorders and found that ASD was the only predictor of reduced *Sensation Seeking* and the strongest predictor (largest regression coefficient and lowest *p*-value) of enhanced *Sensation Avoiding*, while the remaining two sub-domains were more strongly associated with ADHD and affective disorders. The findings are discussed in detail below.

### Sensory symptoms in ASD

In line with a wealth of previous studies that demonstrated an association between sensory processing alterations and ASD (Hazen et al., 2014; Posar & Visconti, 2018), our findings indicate that both continuous and categorical ASD are associated with all AASP sub-domains across individuals, independent of sex and age. More specifically, autistic traits and ASD diagnosis predicted higher scores in all AASP sub-domains except *Sensation Seeking* where the association was negative. Previous studies utilizing the AASP reported a similar association pattern in association with ASD (Chien et al., 2017; Crane et al., 2009). The negative association between ASD and *Sensation Seeking* observed in this and previous studies seems to contradict the observations of “unusual sensory interest,” for instance, reflected by excessive touching or smelling of objects, which is explicitly specified in the DSM-5 as a diagnostic criterion of ASD. We therefore speculate that the latter behaviors differ qualitatively from most of the *Sensation Seeking* behaviors assessed with the AASP, which are predicted to be positively associated with “agreeableness and extraversion” (Dunn, 2001).

### Genetic and environmental impact on the link between ASD and sensory processing alterations

For *Low Registration* and *Sensation Avoiding*, the associations with autistic traits persisted also within MZ twins, indicating that these sub-domains are influenced by non-shared environmental factors, that is, non-genetic factors that make twins dissimilar to each other. All associations had higher p-values within-twin pairs compared to across individuals, likely due to lower power of the within-pair analyses and the impact of familial factors on associations across individuals.

In contrast to all other AASP sub-domains, the within-pair association between autistic traits and *Sensory Sensitivity* was only significant within DZ twins, where the estimate was also three times larger compared to within MZ twins. The lack of an association in the MZ twins can most likely not be explained by a lack of power in the MZ cohort, given that the MZ cohort (*N* = 166) was larger than the DZ cohort (*N* = 103). While the Wald test indicated a difference between MZ and DZ estimate, the results should be interpreted with caution, given that there was a slight overlap between confidence intervals of MZ and DZ cohort estimates.

Our results indicate a genetic impact on the association between *Sensory Sensitivity* and autistic traits, which is in line with the findings of a recent population-based twin study using a classic twin design approach (Taylor et al., 2018). The two studies complement each other adequately methodologically, with the previous study assessing a population-based sample and our study utilizing a comprehensive measure of sensory alterations and a detailed characterization of our twins including gold standard ASD diagnostic instruments.

### Sex and age effects on sensory processing

Female sex predicted higher scores in *Low Registration* and *Sensory Sensitivity*, irrespective of autistic traits and age (please see Supplementary Table 2 for sex and age results). This sex effect was stronger for *Sensory Sensitivity* where being of female sex predicted scoring about half a sample standard deviation higher (4.4 points; for mean values and standard deviations of the four AASP domains, please see Supplementary Table 1 for means and standard deviations). Similarly, in a previous study on adults with and without ASD, females scored higher than males on the AASP total score (Horder et al., 2014). Furthermore, TD females score higher on the Sensory Perception Quotient (Tavassoli et al., 2014) and females with ASD report more life-time sensory issues compared to males (Lai et al., 2011). In contrast, females scored no different from males on the Glasgow Sensory Questionnaire (GSQ, Robertson & Simmons, 2013). Hence, it remains an open question to what extent and in which circumstances females have a different sensitivity to sensory stimuli compared to males.

Higher age was associated with reduced Low Registration in the model with ASD diagnosis as main predictor, and an uncorrected trend in the same direction was observed in the model with autistic traits as the main predictor. Previous studies yielded mixed results regarding the age effects on sensory processing alterations (Ben-Sasson et al., 2019). Of those studies assessing sensory processing with a version of the sensory profile (short or full version, child or adult version), two studies assessing very young individuals (21- and 50-month-old toddlers and 2 to 8-year-old children) did not observe any age effects (McCormick et al., 2016; Rogers et al., 2003), while one study assessing individuals of 3–56 years of age (which is more comparable to our age range of 10–33 years) found a decrease of sensory issues over age across sub-domains and sensory modalities (Kern et al., 2006). Taken together, it is possible that age modulates sensory processing alterations and might also affect the association between sensory processing alterations and ASD. For instance, a meta-analysis found that group differences between individuals with and without ASD in sensory over-responsiveness were strongest at 6–9 years of age (Ben-Sasson et al., 2009).

### How specific are sensory processing alterations related to ASD?

When including three further diagnostic categories in addition to ASD, we found that ASD was the strongest predictor of increased *Sensation Avoiding* and the only diagnosis predicting reduced *Sensation Seeking*, underlining the importance of ASD for these sub-domains of sensory processing alterations. However, having an affective disorder was also a strong predictor of *Sensation Avoiding*, in line with the finding that anxiety traits correlate with *Sensation Avoiding* in people with ASD (Syu & Lin, 2018). *Low Registration* and *Sensory Sensitivity* were most strongly associated with ADHD, corresponding to previous findings of an association between both hypo- and hyper-responsiveness and self-rated ADHD symptoms in adults with ADHD, regardless of concurrent ASD symptoms (Bijlenga et al., 2017). Furthermore, students with ADHD scored similar to students with ASD on the AASP in most sub-domains, further confirming that sensory processing alterations as measured using the AASP are associated with both disorders (Clince et al., 2016). The positive associations between sensory processing alterations and ADHD might be related to the core symptoms of inattention, where higher *Sensory Sensitivity* might reflect a higher distractibility to irrelevant sensory stimuli, whereas increased *Low Registration* might reflect a failure to respond to relevant stimuli, thus resulting in insufficient focus to the task at hand. When modeling each diagnosis in a separate model, many of the associations were stronger as reflected by larger regression estimates and smaller *p*-values, indicating that the different diagnoses partly competed for the same variance in sensory processing alterations. The exception was the sub-domain *Sensation Seeking*, which was solely associated with ASD. Taken together, our findings suggest that sensory processing alterations are not specifically associated with ASD, but that ASD diagnosis might be a particularly strong predictor (compared to other diagnoses) of sub-domains associated with behavioral response patterns aiming to reduce sensory input (more avoiding and less seeking).

### Limitations and future directions

Whether sensory stimuli are perceived as unpleasant and hence sought to be avoided by people with ASD might depend on how predictable they are (Ashburner et al., 2013). Future twin studies should therefore also test the predictability of sensory stimuli as modulator of sensory processing alterations in ASD.

While the sensory profile (different versions, including the AASP) is to date the most widely used assessment tool of sensory processing alterations in individuals with ASD in the average intellectual ability range (DuBois et al., 2017), it also has several limitations. For instance, the sensory profile is unable to distinguish sensory and affective aspects of perception, for example, liking/disliking (Tavassoli et al., 2014) and contains several items with social context, for example, “I move away when others get too close to me.” The inverse correlation of the sensation seeking quadrant with autistic traits observed in this study might indicate that the sensation seeking behaviors assessed with the AASP are not capturing autism-related sensations seeking behaviors very well. This corresponds to results from a recent meta-analysis on sensory processing alterations in individuals with ASD versus TD controls (Ben-Sasson et al., 2019). More specifically, all of the six included studies that utilized the AASP that reported quadrant-specific results found a negative association between ASD and sensation seeking. This is in contrast to most studies using other tools and in contrast to the remaining quadrants where the association with ASD was primarily positive (Ben-Sasson et al., 2019). Moreover, the AASP quadrants generalize across different sensory modalities, in contrast to, for instance, the GSQ, which differentiates seven sensory modalities (Robertson & Simmons, 2013). Hence, we might have overlooked modality-specific effects in our analyses. Taken together, future twin studies should investigate alterations in sensory processing in ASD using also other self-report tools, differentiating between different sensory modalities and avoiding affective language, and in addition more objective measures, free from affective and social aspects, such as sensory detection thresholds.

Our design was optimized for detecting within-pair associations between autistic traits/ASD diagnosis or ADHD diagnosis as predictors of sensory processing alterations. Hence, the associations with diagnostic categories other than ASD and ADHD might have been underestimated. Furthermore, the comparison between MZ and DZ sub-cohorts in our sample provides evidence for the presence of medium sized or large genetic or environmental effects while it is not suitable to estimate the quantitative genetic and environmental contributions within the general population.

## Conclusion

In line with previous studies, our results indicate that both continuous and categorical ASD are associated with four domains of self-reported sensory processing alterations, relating to sensory hyper- or hypo-responsiveness. Our study further provides evidence for a genetically driven link between ASD and increased *Sensory Sensitivity*, while the relationships between ASD and *Low Registration* and *Sensation Avoiding*, respectively, appear to be influenced by non-shared environment. Together, these findings indicate that sensory hyper-reactivity in the sense of experiencing more adverse effects in response to sensory stimuli is part of the ASD genotype, and hence might be an early onset core feature that contributes to other symptoms, while other domains of alterations in sensory processing might be influenced to a larger extent by environmental factors. Finally, the results confirm that sensory processing alterations are not specific to ASD, but that ASD and ADHD predict alterations in different domains most strongly.

## Supplemental Material

sj-pdf-1-aut-10.1177_1362361321991255 – Supplemental material for A co-twin-control study of altered sensory processing in autism Click here for additional data file. Supplemental material, sj-pdf-1-aut-10.1177_1362361321991255 for A co-twin-control study of altered sensory processing in autism by Janina Neufeld, Mark J Taylor, Karl Lundin Remnélius Taylor, Johan Isaksson, Paul Lichtenstein and Sven Bölte in Autism
