# Supplementary material for: A co-twin-control study of altered sensory processing in autism
Source: Autism. 2021 Mar 1;25(5):1422–32. doi: 10.1177/1362361321991255 (PMC8264631; doi:10.1177/1362361321991255)
Supplement: sj-pdf-1-aut-10.1177_1362361321991255 – Supplemental material for A co-twin-control study of altered sensory processing in autism [file sj-pdf-1-aut-10.1177_1362361321991255.pdf]

Supplementary Information

Supplementary Figures

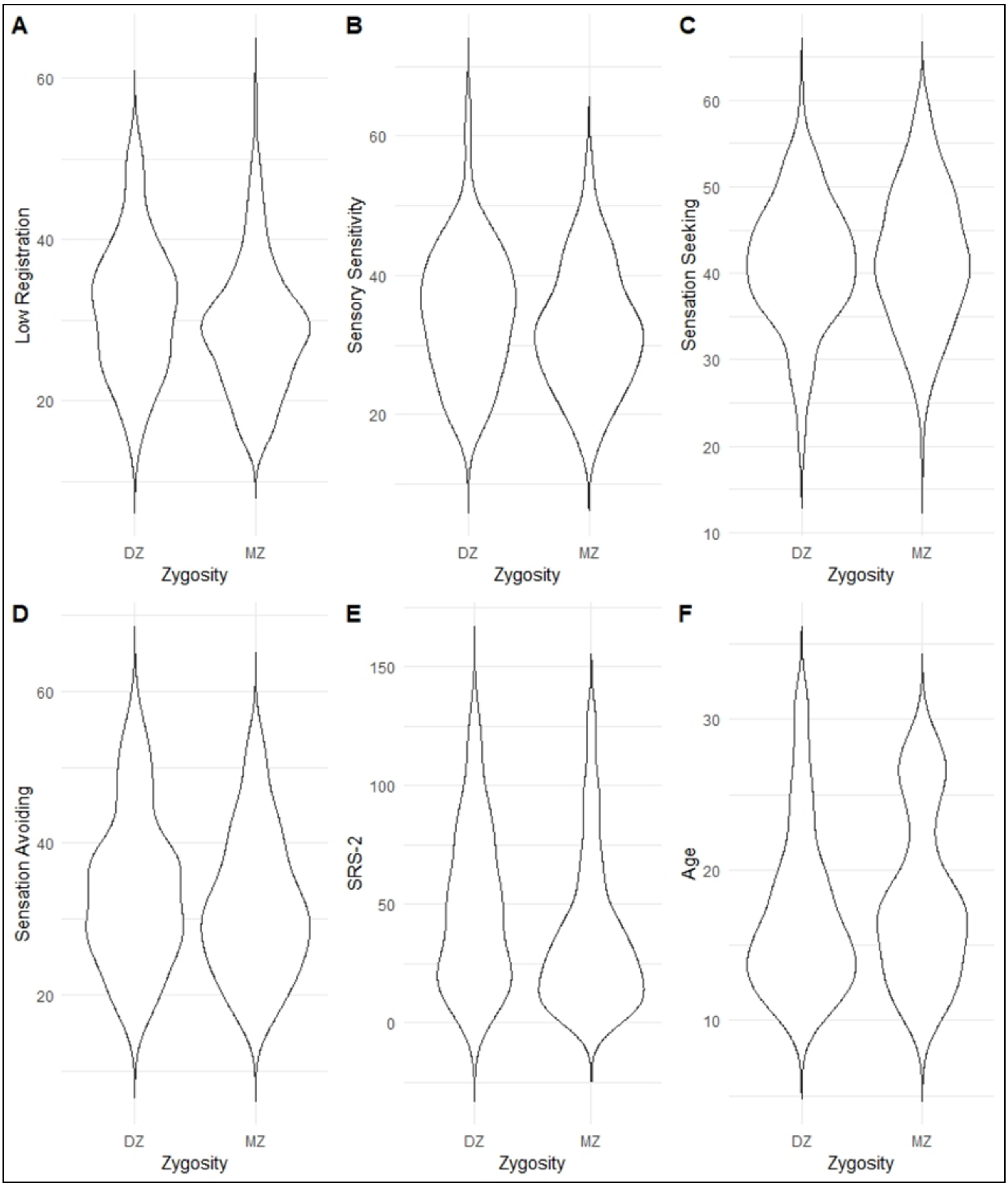

Supplementary Figure 1: Score distributions. *Distribution of the four Adult/Adolescent Sensory Profile quadrant scores, autistic traits (SRS-2) and age in monozygotic (MZ) and dizygotic (DZ) sub-cohorts, visualized using the violin plots function (mirrored density) of ggplot2 in R (geom_violin, Ref: Hintze, J. L., Nelson, R. D. (1998) Violin Plots: A Box Plot-Density Trace Synergism. The American Statistician 52, 181-184).*

Supplementary Tables

***Supplementary Table 1**: Means of sensory processing sub-domains per zygosity group*

AASP sub-domain	*Whole sample*	*MZ*	*DZ*	*t / p*
**Mean Low Registration (SD)**	30.1 (8.3)	29.1 (8.0)	31.7 (4.8)	-2.56 / .01
**Mean Δ Low Registration (SD)**	7.5 (7.2)	6.5 (5.1)	8.2 (7.5)	1.42 / .16
**Mean Sensory Sensitivity (SD)**	33.3 (9.2)	32.4 (8.9)	34.8 (9.5)	-2.05 / .04
**Mean Δ Sensory Sensitivity (SD)**	7.3 (6.3)	6.7 (5.0)	8.6 (7.8)	1.56 / .12
**Mean Sensation Seeking (SD)**	41.6 (7.7)	41.9 (9.7)	41.2 (7.4)	.71 / .48
**Mean Δ Sensation Seeking (SD)**	7.3 (6.0)	6.7 (5.5)	8.3 (6.7)	1.45 /.15
**Mean Sensation Avoiding (SD)**	32.5 (9.6)	31.7 (9.3)	33.8 (10)	-1.74 / .08
**Mean Δ Sensation Avoiding (SD)**	7.4 (7.0)	6.3 (5.7)	8.6 (7.7)	1.81 / .07

***Note.** Means and standard deviations (SD) of the four Adult/Adolescent Sensory Profile sub-domains for the whole sample and monozygotic (MZ) and dizygotic (DZ) sub-cohorts. Δ = within-twin pair differences, t/p = t-values and p-values from t-tests comparing MZ and DZ sub-cohorts. Note that within-pair differences did not differ between zygosity groups for any sub-scale.*

***Supplementary Table 2:** Results for covariates from models across individuals*

	Sex			*Age*		
	b [95% CI]	*SE*	*Z / p*	*b [95% CI]*	*SE*	*Z / p*
**Model with autistic traits as main predictor**						
Low Registration	**2.68 [.61-4.75]**	**1.06**	**2.53 / .01**	-.20 [-.38 -.01]	.10	-2.08 / .04
Sensory Sensitivity	**4.42 [2.08, 6.76]**	**1.20**	**3.70 / 2.2e^{-4}**	.03 [-.21, .27]	.12	.26 / .80
Sensations Seeking	2.08 [.19, 3.97]	.97	2.15 / .03	.08 [-.11, .28]	.10	.84 / .40
Sensation Avoiding	2.57 [.11, 5.03]	1.26	2.05 / .04	.18 [-.06, .42]	.12	1.48 / .14
**Model with ASD diagnosis as main predictor**						
Low Registration	**2.71 [.59, 4.83]**	**1.08**	**2.51 / .01**	**-.33 [-.52, -.15]**	**.09**	**-3.59 / 3.3e^{-4}**
Sensory Sensitivity	**4.44 [2.01, 6.88]**	**1.24**	**3.58 / 3.5e^{-4}**	-.12 [-.35, .12]	.12	-.96 / .34
Sensations Seeking	2.01 [.18, 3.84]	.93	2.16 / .03	.14 [-.04, .32]	.09	1.55 / .12
Sensation Avoiding	2.63 [.00, 5.27]	1.35	1.96 / .05	-.02 [-.25, .22]	.12	-0.14 / .89

***Note**. Results for the covariates individuals (sex and age) included in the model across for the different models where AASP domains (rows) were the outcomes and autistic traits or ASD diagnosis the main predictor. b = regression coefficient, 95%CI = 95% confidence interval, SE = standard error. Z = Z statistics. Significant outcomes are printed in* **BOLD.**

Supplementary Table 3: *Standardized regression coefficients as effect size estimates*

Model	***Autistic traits β [95% CI]***	***ASD diagnosis β [95% CI]***
Low Registration		
across unadjusted	.40 [.27, .53]	.65 [.34, .95]
across adjusted	.37 [.24, .50]	.59 [.29, .89]
within-pairs (MZ)	.47 [.27, .66]	-
within-pairs (DZ)	.50 [.23, .77]	-
Sensory Sensitivity		
across unadjusted	.31 [.18, .45]	.47 [.11, .82]
across adjusted	.34 [.20, .48]	.47 [.14, .81]
within-pairs (MZ)	.17 [-.03, .36]	-
within-pairs (DZ)	.52 [.25, .79]	-
Sensation Seeking		
across unadjusted	-.29 [-.39, -.18]	-.85 [-1.10, -.59]
across adjusted	-.25 [-.37, -.14]	-.80 [-1.06, -.54]
within-pairs (MZ)	-.23 [-.46, -.01]	-
within-pairs (DZ)	-.18 [-.44, .08]	-
Sensation Avoiding		
across unadjusted	.43 [.31, .55]	.84 [.51, 1.17]
across adjusted	.48 [.36, .60]	.85 [.54, 1.17]
within-pairs (MZ)	.26 [.10, .42]	-
within-pairs (DZ)	.51 [.24, .77]	-

Note. *Standardized regression coefficients and their 95% confidence intervals (95 % = CI) from linear regressions across individuals (adjusted and unadjusted) and within-twin pairs (MZ = monozygotic, DZ = dizygotic) for* ***autistic traits*** *and* ***ASD diagnosis*** *(middle and right column) as main predictors of sensory processing. The standardized estimates indicate how many standard deviations (or what proportion of a standard deviation) change in sensory processing alterations is associated with an increase of one standard deviation in autistic traits or having an ASD diagnosis.*
